# The NLstart2run study: health effects of a running promotion program in novice runners, design of a prospective cohort study

**DOI:** 10.1186/1471-2458-13-685

**Published:** 2013-07-26

**Authors:** Bas Kluitenberg, Marienke van Middelkoop, Ron L Diercks, Fred Hartgens, Evert Verhagen, Dirk-Wouter Smits, Ida Buist, Henk van der Worp

**Affiliations:** 1 Center for Sports Medicine, University of Groningen, University Medical Center Groningen, Hanzeplein 1, 9700 RB, Groningen, The Netherlands; 2 Department of General Practice, Erasmus MC Medical University, Rotterdam, The Netherlands; 3 Departments of Epidemiology and Surgery, Research School CAPHRI, Maastricht University Medical Centre+, and Sports Medicine Centre Maastricht, Maastricht, The Netherlands; 4 Department of Public and Occupational Health, EMGO+ Institute for Health and Care Research VU University Medical Center, Amsterdam, The Netherlands; 5 Department of Rehabilitation, Nursing Science and Sports, University Medical Center Utrecht, Utrecht, The Netherlands

## Abstract

**Background:**

Running is associated with desirable lifestyle changes. Therefore several initiatives have been undertaken to promote running. Exact data on the health effects as a result of participating in a short-term running promotion program, however, is scarce. One important reason for dropout from a running program is a running-related injury (RRI). The incidence of RRIs is high, especially in novice runners. Several studies examined potential risk factors for RRIs, however, due to the often underpowered studies it is not possible to reveal the complex mechanism leading to an RRI yet.

The primary objectives are to determine short- and long-term health effects of a nationwide “Start to Run” program and to identify determinants for RRIs in novice runners. Secondary objectives include examining reasons and determinants for dropout, medical consumption and economical consequences of RRIs as a result of a running promotion program.

**Methods/design:**

The NLstart2run study is a multi-center prospective cohort study with a follow-up at 6, 12, 24 and 52 weeks. All participants that sign up for the Start to Run program in 2013, which is offered by the Dutch Athletics Federation, will be asked to participate in the study.

During the running program a digital running log will be completed by the participants every week to administer exposure and running related pain. After the running program the log will be completed every second week. An RRI is defined as any musculoskeletal ailment of the lower extremity or back that the participant attributed to running and hampers running ability for at least one week.

**Discussion:**

The NLstart2run study will provide insight into the short- and long-term health effects as a result of a short-term running promotion program. Reasons and determinants for dropout from a running promotion program will be examined as well. The study will result in several leads for future RRI prevention and as a result minimize dropout due to injury. This information may increase the effectiveness of future running promotion programs and will thereby contribute positively to public health.

**Trial registration:**

The Netherlands National Trial Register NTR3676. The NTR is part of the WHO Primary Registries.

## Background

Several initiatives have been undertaken to encourage people to start running. In the Netherlands a nationwide supervised running promotion program called Start to Run is offered by the Dutch Athletics Federation, which attracts over 8,000 novice runners annually. So far, the exact health benefits of a 6-week running promotion program and reasons for dropout have barely been examined (Van Merode T, Vasse R, Twellaar M, Hartgens F: Positive healthy lifestyle changes: high compliance and adherence to exercise in training programmes for novice sporters (start-to-run and start-to-walk); 6 weeks and 6 months follow-up, submitted).

Running is associated with desirable lifestyle changes such as weight loss, smoking cessation and improvement in cardio respiratory fitness and mental health [1-3].The health benefits associated with a short-term promotion program for novice runners, however, have not yet been examined. Effectiveness of such a program increases the possibilities to improve public health.

Preliminary dropout from a running promotion program hinders a shift from extrinsic motivation to intrinsic motivation, which is important in becoming a regular exerciser [4]. An important dropout reason from these programs is the occurrence of a running-related injury (RRI) [1]. Consequently, sustaining an RRI is associated with failure to start and maintain a physically active lifestyle [5]. Moreover, research has shown that especially novice runners are at a high risk for sustaining an RRI [6-8]. Prevention of RRIs in novice runners is therefore of particular importance. Prevention of RRIs in novice runners participating in a running promotion program is especially important to minimize medical costs associated with RRIs and to maximize the positive health effects by maintaining an active lifestyle [1,9]. From this perspective it is also important to identify other reasons besides RRIs for dropout from a start-to-run program.

Several studies tried to identify determinants for RRIs. One of the reasons that the most important risk factors for RRIs have not yet been identified might be related to the often-underpowered studies performed. Since the development of an RRI results from a complex interaction between both intrinsic and extrinsic risk factors, a multivariate analysis is essential for identifying risk factors for RRIs [10,11]. Multivariate methods are susceptible to producing problematic results if too few RRIs are available relative to the number of risk factors analyzed in the model. As a result, several studies that used this type of analysis first examined the independent link of potential risk factors with RRIs and only factors univariately associated with an RRI were entered into the multivariate model [7,12-14]. To account for the multifactorial nature of RRIs this univariate selection of potential risk factors is undesirable [15]. A large cohort study would increase the number of factors that can be entered into a multivariate model and therefore reduce the need for a univariate selection, hence it is needed for identification of risk factors for general and specific RRIs which is important for the development of preventive measures.

The primary objectives of the NLstart2run study are therefore to determine short- and long-term health effects of a nationwide “Start to Run” program and to identify determinants for RRIs in novice runners. This information is important for developing successful running promotion programs in the future [16]. Secondary objectives are to determine reasons and determinants for dropout from a supervised running promotion program, and to identify the course and medical consumption of injured runners and the associated costs. In this article we describe the protocol of the prospective cohort study NLstart2run.

## Methods/design

### Study design

**Figure 1:**
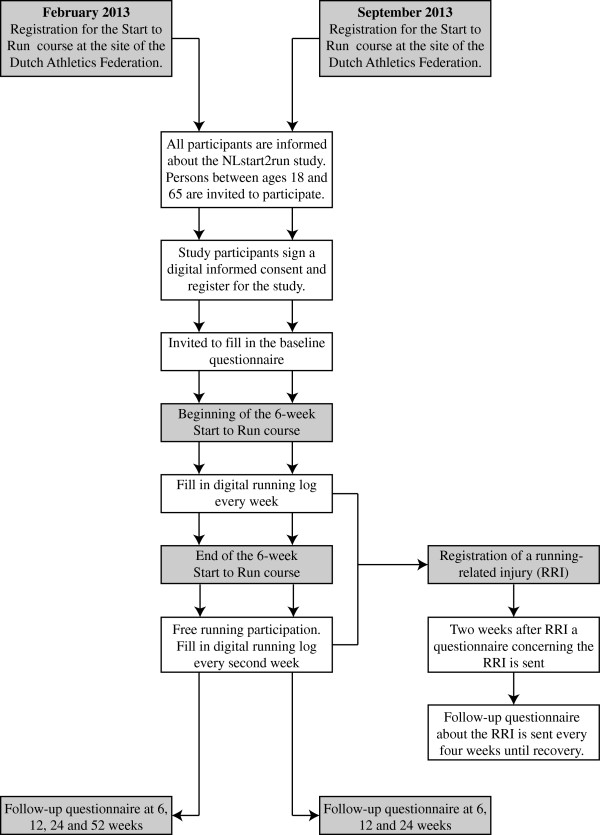
Flowchart of the NLstart2run study.

The NLstart2run study is a multi-center prospective cohort study with a follow-up at 6, 12, 24 and 52 weeks, as can be seen in Figure 1. The study design, procedures and informed consent procedure were approved by the Medical Ethics Committee (no. 2012/350) of the University Medical Center Groningen (UMCG), the Netherlands. The trial is registered in the Netherlands Trial Registry (NTR3676).

### Study population

All participants that sign up for the 6-week Start to Run (STR) program of the Dutch Athletics Federation in 2013 will be asked to participate in the NLstart2run study. The STR course is organized twice a year and starts in March and September. The STR participants will be recruited by the Dutch Athletics Federation via national and local media. Additional information about the study and procedures is sent to all STR participants. Participants can register for the study after signing a digital informed consent.

#### ***Inclusion & exclusion criteria***

Participants of the STR program who are aged between 18 and 65 are eligible for inclusion. Participants will be excluded if there are absolute contraindications for vigorous physical activities according to the American College of Sports Medicine ACSM) guidelines [17], or in case of unwillingness to keep a running log.

### The STR training program

The STR training program consists of three training sessions per week, one group training session supervised by a licensed athletic trainer and two individual training sessions. Each training session begins with a 15-minute warm-up and finishes with a 15-minute cool-down that consist of walking, stretching and relaxation exercises. The core of the training includes running program in which both running duration and intensity will be gradually increased. The STR program aims to prepare participants in 6 weeks for a 20-minute run without breaks. After the 6-week STR program, participants can choose to continue running in a way they prefer.

### Injury definition

In the current study a running-related injury (RRI) is defined as any musculoskeletal ailment of the lower extremity or back that the participant attributed to running and hampers running ability for at least one week [14,18]. Hampering of running can be either a reduction in running speed, distance or duration, or an inability to run, both as a result of running-related pain.

### Sample size

Based on previous studies on RRIs among novice runners, an injury incidence of 20% is expected [7,14,18]. Taking into account a dropout rate of 20% [6,7,14,18,19], the inclusion of 6,000 participants will lead to approximately 4,800 participants at follow-up. With an RRI incidence of 20%, a total of 960 RRIs are expected. Taking into account the need of a minimum of 10 RRIs per risk factor analyzed in a multivariate model, this is sufficient for accounting for the multivariate nature of RRIs [15,20].

### Measurements

#### ***Baseline questionnaire***

The five-part baseline questionnaire will be administered online after participants give informed consent. Demographics, anthropometrics, and other personal characteristics are covered in part 1. Self-reported body height and weight will be used to calculate BMI (weight (kg) / height^2^ (m)).

Part 2 covers information on previous running and sports participation [21,22]. Also information about previous injuries during running (“Have you ever had a running injury?”) and musculoskeletal complaints in other sports (“Have you ever had complaints in your bones, joints or muscles and tendons during sports and exercise?”) are obtained per anatomical site. Information on footwear and insoles is obtained in the second part as well.

Physical activity during daily life is assessed in part 3 with the Short Questionnaire to Assess Health-enhancing physical activity (SQUASH). The SQUASH was designed to give an indication of habitual activity level during an ordinary week. The SQUASH has been tested for validity and reliability with an accelerometer as a criterion measure in a general adult population and has turned out to be reliable and valid [23,24].

In part 4, the Behavioral Regulation in Exercise Questionnaire-2 (BREQ-2) is used to measure motivation toward exercise in general. The BREQ-2 assesses a motivation and external, identified, interjected and intrinsic regulations, and showed sufficient validity in adults [25].

In the final part physical, mental and social health is measured. Perceived health is assessed with the Dutch version of the RAND-36 Health Survey (RAND-36), which was translated from the standardized SF-36 Health Survey [26,27]. The RAND-36 is a validated and reliable measure [26]. Mastery as part of mental health is measured using the 7-item scale of Pearlin and Schooler [28]. Physical health is measured with a 5-item scale on high prevalent health complaints, and social health is measured with the shortened 6-item scale for loneliness [29,30].

#### ***Follow-up questionnaires***

The runners starting with the STR program in March 2013 will receive four follow-up questionnaires at 6, 12, 26 and 52 weeks after beginning with the program. The group of runners who start in September will only receive three follow-up questionnaires at 6, 12 and 26 weeks. In the follow-up questionnaires continuation and reasons for discontinuation of running will be monitored. Participation in other sports will also be monitored in the follow-up questionnaires. Besides questions about continuation of running or other sport activities, all follow-up questionnaires also measure physical activity (SQUASH), motivation toward exercise (BREQ-2), and physical and mental health (RAND-36, 7-items scale of Pearlin and Schooler, 5-item scale on high prevalent health complaints, and shortened 6-item scale for loneliness).

#### ***Web-based training log during the 6-week STR program***

During the 6-week STR program a weekly training log is sent to the participants. For each training session, data on running exposure (frequency and duration), running surface, perceived exertion and pain is registered. Weekly information on other sports activities (type of sports and exposure that week) is obtained from the training log. When participants do not enter their digital training log after five days, an e-mail reminder is sent automatically.

Perceived exertion will be assessed using Borg’s Ratings of Perceived Exertion (RPE) scale. The RPE scale is widely used in exercise science and sports medicine to monitor or prescribe levels of exercise intensity. Borg’s RPE scale has shown to be a valid measure of exercise intensity [31].

Pain will be measured by registering anatomical site of the body and severity of pain for each training session. A mannequin will be shown to identify the anatomical site of the running-related pain. By clicking on the anatomical site, the same spot will be pointed red. Severity of pain is additionally subdivided into pain without limitations, pain that causes a restriction in running, and pain which makes running impossible. To classify the severity of pain, a Visual Analog Scale (VAS) is used. When a training session is skipped, the reason for it will be asked (running-related pain, other pain, illness, motivation, or other reason). When running-related pain is the reason, anatomical site and severity of pain will be obtained.

In addition to the pain registration obtained for each training session, the development and progression of overuse injuries is also monitored with a new method as proposed by Clarsen et al. [32]. This method is designed to monitor overuse problems for predefined anatomical locations separately. In the current study, however, this is not ideal because all injury locations are of interest. The proposed method has thus been modified: instead of monitoring pain for each predefined anatomical location, general data is collected and the specific anatomical location is asked for afterwards.

#### ***Web-based training log after the 6-week STR program***

After the 6-week STR program, a digital training log is sent to the participants every two weeks. This training log is a simplified version of the log that was used during the first six weeks. Information on running exposure, running surface, running-related pain and other sports activities is registered per week instead of at each training session. In this simplified training log, pain registration and monitoring of overuse injuries is similar to the methods used in the first six weeks. Participants who quit running after the STR program can indicate so, and only have to complete the information on other sports activities.

#### ***Questionnaire for injured runners***

When an RRI is registered in the web-based training log, a questionnaire will be automatically sent to the participant after two weeks. This questionnaire is used to specify medical/paramedical treatment, the order of treatments, type and frequency of the treatment, medical aids, and absence from work, school or training due to the RRI. Injured runners are asked to specify the injury location, if the injury is new or recurrent, what structure is injured (e.g. muscle, tendon, bone or ligament), and the injury mechanism (e.g. strain, sprain, rupture, dislocation, contusion, inflammation or overuse). When the runner is seen by a professional, a diagnosis will be asked. Until recovery from the RRI, every four weeks a follow-up questionnaire on medical/paramedical consumption and absence from work, school or training is sent to the injured runner.

To validate the RRI registration method that is used in this study, approximately 10% of the subjects who sustain an RRI will be contacted by a sports physician. The sports physician will perform a standard physical examination and will then complete the abovementioned RRI questionnaire regarding location, structure, and mechanism of the injury.

#### ***RRI cost analysis***

From the data derived from the RRI questionnaire and subsequent follow-up questionnaires, direct and indirect costs will be calculated. Costs will also be calculated from a societal perspective. Direct healthcare costs will include costs resulting from medical/paramedical treatment and/or medical aids. Indirect costs will include costs resulting from absence from paid work or unpaid work, as well as leisure time lost. Dutch guideline prices will be used to value resource use [33]. Direct costs of hospital treatment will be estimated on the basis of standard prices from the Health Care Insurance Board [34]. The costs of medication and medical aids are estimated on the basis of prices recommended by the Royal Dutch Society of Pharmacy [35] and the Health Care Insurance Board [34,36]. Costs of loss of productivity due to absenteeism from paid or unpaid work will also be included. Costs of absenteeism from paid work are estimated using the friction cost approach with a friction period of four months and based on the mean age- and sex-specific income of the Dutch population. [33]. Costs of productivity loss attributable to unpaid work, such as study and household work, will be estimated at a shadow price of EUR 8.78/hour. All prices are standardized to the year 2012 and will be adjusted for inflation [36]. Total costs will be estimated for each injured athlete by multiplying resource data by cost prices. Total, direct and indirect costs will be calculated by adding costs per category of utilization of healthcare resources.

### Statistical analyses

Incidence of RRIs will be calculated for all participants and for male and female participants separately as the number of new injuries reported per 1000 hours of running exposure. Exposure time (in hours of running exposure) will be calculated from the time the participant started the running program until an RRI is reported (injured runners) or until the end of the program (non-injured runners).

Descriptive data will be presented as means (± s.d.) and frequency distributions.

For identification of risk factors for RRIs only data from the baseline questionnaire and the running log will be used. Data of the follow-up questionnaires will not be used for this purpose, because these data do not contribute to a pre-running risk-profile. It is possible for a single subject to sustain multiple RRIs during the study period. In this case only the occurrence of the first RRI will be used for analysis. Potential risk factors for RRI will be entered into a multivariate Cox regression prediction model. Hazard ratios and the corresponding 95% confidence intervals will be calculated for the factors associated with RRI.

Data of the baseline and follow-up questionnaires will be compared with a repeated-measures ANOVA to examine the health effects of participating in a supervised running program. Reasons for dropout from the start-to-run program will be described and determinants for dropout will be analyzed by multivariate logistic regression models. Course and medical consumption and the associated direct and indirect costs will be described for injured runners.

Missing data will be completed by multiple imputation using the Multiple Imputation by Chained Equations (MICE) procedure, a technique in which missing values are replaced based on estimated relations in the dataset [37]. Ten multiple imputed datasets are generated, whereupon the results of those ten multiple imputed datasets will be combined using the rules given by Rubin [37].

## Discussion

The extensive baseline questionnaire and follow-up questionnaires will provide insight into the short- and long-term health effects of participation in a 6-week running promotion program. Perceived health, physical activity in daily life and motivation to exercise will be monitored over time and thereby describe the effects of a running program for novice runners. Sports participation during and after the 6-week STR course will be monitored. Reasons and determinants for discontinuation for both running and sports will be examined as well. These findings may reduce dropout rates from future running or sports promotion programs and increase adherence to sports during and after a program.

The NLstart2run study will result in several leads for future RRI prevention programs. The large sample size and the extensive baseline measurements will result in ample information on determinants for RRIs in novice runners. As a result of the large group of participants, it will be possible to study risk factors for specific RRIs or in specific subgroups. These findings will result in new information regarding modifiable risk factors that can be applied in future running promotion programs to minimize dropout due to injury.

As a result of the NLstart2run study, valuable information on health effects and RRI prevention for novice runners will be gained. Moreover, medical consumption and consequences of an RRI, as well as reasons and determinants for discontinuation and dropout from a running promotion program will be examined. This information can be used for the implementation of more effective running promotion programs, thus making a positive contribution to public health.

## Competing interests

The authors declare that they have no competing interests.

## Authors’ contributions

IB, MM, RD conceived of the idea and obtained funding for the study. BK is the study investigator, wrote the article and will be responsible for data acquisition. FH, EV, DWS, HW provided advice on the study design and contributed to the content of the article. RD, MM and HW coordinate the trial. All authors read and approved the final manuscript.

## Pre-publication history

The pre-publication history for this paper can be accessed here:

http://www.biomedcentral.com/1471-2458/13/685/prepub
